# Supplementary material for: Assessing the impact of online postal self-sampling for sexually transmitted infections on health inequalities, access to care and clinical outcomes in the UK: protocol for ASSIST, a realist evaluation
Source: BMJ Open. 2022 Dec 14;12(12):e067170. doi: 10.1136/bmjopen-2022-067170 (PMC9756155; doi:10.1136/bmjopen-2022-067170)
Supplement: Supplementary data [file bmjopen-2022-067170supp001.pdf]

ASSIST service user topic guide

The interview is conducted flexibly, using language familiar to the participant

Introduction to interview

- Interviewer introduces self; explains purpose of interview, why participant has been invited, and how interview will run
- Interviewer reassures participant about confidentiality of interview, and interest in their views (no right or wrong answers)
- Interviewer explains interview should take around 60 minutes and explains key topics

Internet and smartphone use

How they go online (e.g. wifi, fixed internet, mobile data). What they access online, including health information and healthcare online.

Previous experiences of accessing sexual health services

Talk through their experiences of accessing sexual health services, including what worked well and what could have been improved.

Experience of accessing OPSS [only if known experience from screening questionnaire]

Talk through their first experience of accessing OPSS, covering each component of the user journey (exploring expectations, emotional responses, what happened etc).

If they have accessed OPSS more than once, ask about experiences of accessing services since then exploring the same points.

Knowledge and views of OPSS [if no known experience from screening questionnaire]

Talk through their awareness of OPSS (exploring thoughts, whether they would consider it etc).

Explore each component of the user journey for OPSS in their area.

Scenarios [for all whether experience or no experience of OPSS]

Interviewer introduces concept of scenarios for using OPSS vs clinic-based services and invites interviewee to talk through thought process for different scenarios, focusing on how the person might choose to get tested and why.

Ending

Thank participant for their time; check if any questions / OK to contact them for clarification.

Provide with information about support services that they can access.

ASSIST study, 140431, IRAS 295506, REC Reference 21/SC/0223, Service user interview topic guide, 1-page Version 1.0 [08/06/2021]
