# Supplementary material for: Assessing the impact of online postal self-sampling for sexually transmitted infections on health inequalities, access to care and clinical outcomes in the UK: protocol for ASSIST, a realist evaluation
Source: BMJ Open. 2022 Dec 14;12(12):e067170. doi: 10.1136/bmjopen-2022-067170 (PMC9756155; doi:10.1136/bmjopen-2022-067170)
Supplement: Supplementary data [file bmjopen-2022-067170supp002.pdf]

ASSIST

Assessing the impact of online self-sampling for STIs & HIV

ASSIST staff & stakeholder (adoption) topic guide

The interview is conducted flexibly

Introduction to interview

- Interviewer explains purpose of interview and how interview will run
- Interviewer reassures participant about confidentiality of interview
- Interviewer explains interview will be tailored according to participant's role

You and your role

Participant invited to describe their role(s) in the service [this information is used to decide which questions to use]

Introduction of OPSS / fit with existing values and ethos

Talk through first considering / first hearing about using OPSS in their service (exploring what they and their colleagues thought, the decision to introduce it, its fit with existing ethos/culture, and the introduction of OPSS)

Understanding of OPSS in your context

How OPSS is delivered in their area, how this differs from what they did before, advantages and disadvantages of face-to-face vs remote sexual healthcare delivery

Impacts of OPSS on you, service, team

The process of implementation and the impact that OPSS may have had on clinical work (exploring processes to support staff to deliver OPSS, change in skills, impact on division of work / relationships, anything that made implementing OPSS hard or easy, interactions with patients / consultations)

Reflections and changes

Changes that have been made, changes to the context of delivering OPSS, aspects that have worked well / could have been done better, has it been worth it

Closing

Anything the participant would like to raise?

Thank participant for their time

ASSIST study, 140431, IRAS 295506, REC Reference 21/SC/0223, Staff & stakeholder interview topic guide (adoption), 1-page
Version 1.0 [08/06/2021]
