# Supplementary material for: Assessing the impact of online postal self-sampling for sexually transmitted infections on health inequalities, access to care and clinical outcomes in the UK: protocol for ASSIST, a realist evaluation
Source: BMJ Open. 2022 Dec 14;12(12):e067170. doi: 10.1136/bmjopen-2022-067170 (PMC9756155; doi:10.1136/bmjopen-2022-067170)
Supplement: Supplementary data [file bmjopen-2022-067170supp003.pdf]

Assessing the impact of online self-sampling for STIs & HIV

ASSIST staff & stakeholder (delivery) topic guide

The interview is conducted flexibly

Introduction to interview

- Interviewer explains purpose of interview and how interview will run
- Interviewer reassures participant about confidentiality of interview
- Interviewer explains interview will be tailored according to participant's role

You and your role

Participant invited to describe their role(s) in the service

Introduction of OPSS / fit with existing values and ethos

Talk through first hearing about using OPSS in their service (exploring what they and their colleagues thought, the decision to introduce it, its fit with existing ethos/culture)

Understanding of OPSS in your context

How OPSS is delivered in their area, how this differs from what they did before, when OPSS is recommended, personal preference for face-to-face vs remote, different people it works 'better' for (or not)

Impacts of OPSS on you, service, team

The impact that OPSS has had on work (exploring interactions with patients / consultations, interactions with other clinic staff, change in skills, impact on division of work / relationships, anything that made implementing OPSS hard or easy, processes to support delivery of OPSS)

Reflections and changes

Changes that have been made, changes to the context of delivering OPSS, aspects that have worked well / could have been done better, has it been worth it

Closing

Anything the participant would like to raise?

Thank participant for their time

Gibbs J, *et al. BMJ Open* 2022; 12:e067170. doi: 10.1136/bmjopen-2022-067170
